# Mechanisms of human telomerase reverse transcriptase (h*TERT*) regulation: clinical impacts in cancer

**DOI:** 10.1186/s12929-018-0422-8

**Published:** 2018-03-12

**Authors:** Ricardo Leão, Joana Dias Apolónio, Donghyun Lee, Arnaldo Figueiredo, Uri Tabori, Pedro Castelo-Branco

**Affiliations:** 1 Division of Urology, Department of Surgery Princess Margaret Cancer Centre, University Health Network, 610 University Ave 3-130, Toronto, ON M5G 2M9 Canada; 2 Arthur and Sonia Labatt Brain Tumor Research Center, The Hospital for Sick Children, University of Toronto, 555 University Avenue, Toronto, ON M5G 1X8 Canada; 3 Faculty of Medicine, University of Coimbra, R. Larga, 3004-504 Coimbra, Coimbra Portugal; 4 Department of Urology, Coimbra University Hospital, Coimbra, Portugal; 5 Regenerative Medicine Program, Department of Biomedical Sciences and Medicine, University of Algarve, Edifício 2 – Ala Norte, 8005-139 Faro, Portugal; 6 Centre for Biomedical Research (CBMR), University of Algarve, Faro, Portugal; 7 Algarve Biomedical Center, Campus Gambelas, Faro, Portugal; 8 Division of Haematology/Oncology, The Hospital for Sick Children, 555 University Avenue, Toronto, M5G 1X8ON Canada

**Keywords:** Telomeres, Telomerase, Telomerase regulation, Cancer biomarkers

## Abstract

**Background:**

Limitless self-renewal is one of the hallmarks of cancer and is attained by telomere maintenance, essentially through telomerase (h*TERT*) activation. Transcriptional regulation of h*TERT* is believed to play a major role in telomerase activation in human cancers.

**Main body:**

The dominant interest in telomerase results from its role in cancer. The role of telomeres and telomere maintenance mechanisms is well established as a major driving force in generating chromosomal and genomic instability. Cancer cells have acquired the ability to overcome their fate of senescence via telomere length maintenance mechanisms, mainly by telomerase activation.

h*TERT* expression is up-regulated in tumors via multiple genetic and epigenetic mechanisms including h*TERT* amplifications, h*TERT* structural variants, h*TERT* promoter mutations and epigenetic modifications through h*TERT* promoter methylation. Genetic (h*TERT* promoter mutations) and epigenetic (h*TERT* promoter methylation and miRNAs) events were shown to have clinical implications in cancers that depend on h*TERT* activation. Knowing that telomeres are crucial for cellular self-renewal, the mechanisms responsible for telomere maintenance have a crucial role in cancer diseases and might be important oncological biomarkers. Thus, rather than quantifying *TERT* expression and its correlation with telomerase activation, the discovery and the assessment of the mechanisms responsible for *TERT* upregulation offers important information that may be used for diagnosis, prognosis, and treatment monitoring in oncology. Furthermore, a better understanding of these mechanisms may promote their translation into effective targeted cancer therapies.

**Conclusion:**

Herein, we reviewed the underlying mechanisms of h*TERT* regulation, their role in oncogenesis, and the potential clinical applications in telomerase-dependent cancers.

## Background

Replicative capacity is one of the most critical features in cancer cells, which is attained by telomere maintenance [1]. Telomeres protect the ends of chromosomes from degradation and end-to-end fusions, contributing to genomic stability [1, 2]. Telomerase, a specialized DNA polymerase, is responsible for telomere maintenance in the majority of human cancers, but its activity is absent in most normal somatic tissues. This differential role makes telomerase and its regulatory mechanisms attractive cancer biomarkers with relevant implications in clinical practice [3].

### Telomeres and telomerase

**Fig. 1:**
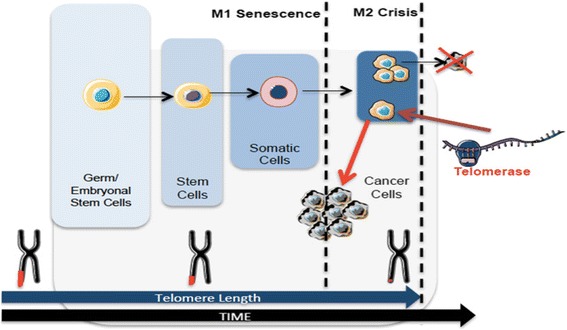
Telomere length dynamics in different cells over time. Telomeres shorten over time. Germ cells and embryonic stem cells have long telomeres that are maintained by telomerase activity. Stem cells have shorter telomeres and somatic cells even shorter. After multiple cell divisions these cells achieve a senescence state (M1). At M2 stage cells enter crisis due to their short telomeres that lead to chromosomal and genomic instability resulting in apoptosis. Cancer cells escape from crisis through telomerase activation, reacquire longer telomeres and unlimited self-renewal capacity

Telomeres are the nucleoprotein complexes located at the ends of eukaryotic chromosomes. Telomere structure was discovered by Muller and Meier in 1938. Telomeres consist of 5 to 20kb of repeating hexanucleotide DNA sequence TTAGGG (telomeric DNA) [4–6]. Telomeric DNA repeats are followed by a terminal 3´G-rich single-stranded overhang forming a telomeric loop (T-loop) that provides 3´end protection [7, 8]. Telomeric DNA is associated with the shelterin protein complex and together they protect chromosomal ends and maintain genomic and chromosomal integrity by preventing nucleolytic degradation, unnecessary recombination, and inter-chromosomal fusions [7, 9, 10]. The shelterin complex consists of a group of six telomere-specific proteins; telomeric repeat binding factor 1 and 2 (TERF1, TERF2) and protection of telomeres protein 1 (POT1) interact directly with TTAGGG repeats. These proteins are interconnected with three others: TERF1 Interacting Nuclear Factor 2 (TINF2), tripeptidyl-peptidase 1 (TPP1), and repressor activator protein 1 (RAP1) [7, 8, 11]. Telomeric DNA is masked with shelterin protective caps and these complexes enable DNA damage repair (DDR) machinery to distinguish telomeric DNA from genomic DNA damage [12, 13]. Throughout cellular lifespan, telomeric DNA is shortened after each replicative cycle due to the “end-replication problem”, oxidative damage, age, and lifestyle (including diet, smoking, professional environment and stress) [14–16]. Telomere shortening leads to a stage of cell growth arrest. At this stage (M1), DNA damage signalling and cellular senescence are triggered which constitutes a crucial protective mechanism that prevents progression to an oncogenic state [10, 17]. However, in some cases, cells surpass this senescence state (avoiding important cell cycle checkpoints provided by p16^INK4a^, TP53 and Rb) and enter a crisis state (M2) [17]. At this point, cells have very short telomeres and their chromosomal ends fuse, leading to chromosome bridge-breakage-fusion cycles, genomic instability, and eventually cell apoptosis [17]. However, in rare situations, cells may acquire the ability to continuously divide which may promote malignant transformation (Fig. 1). This process of unlimited self-renewal is mediated by telomerase that maintains or lengthen telomeres promoting cellular immortalization process [1, 3, 17].

Telomerase was discovered in 1985, as an enzyme capable of extending telomeric repeat sequences; and in 1989, telomerase activity was reported for the first time [18–20]. However, the protein component of telomerase was only identified and functionally characterized in 1997, more than a decade after its discovery [21]. This enzyme consists of a large ribonucleoprotein complex responsible for progressive synthesis of telomeric DNA repeats. Telomerase is a DNA polymerase that consists of two different subunits: a functional catalytic protein subunit called human telomerase reverse transcriptase (h*TERT*) encoded by the *TERT* gene, positioned at chromosome 5p15.33; and a RNA component known as human telomerase RNA component (h*TERC* or h*TR*), encoded by the *TERC* gene on chromosomal region 3q26 [22–24]. Other proteins including Pontin, Reptin, Gar1, Nhp2, and Tcab1 were shown to be associated with the telomerase core complex and required for proper telomerase assembly and recruitment to chromosomes [25, 26]. Dyskerin and telomerase protein component (TEP1) have an important role in stabilizing the telomerase complex [27, 28]. Es1p and Es3p are additional protein subunits (Ku heterodimer) involved in assembly and maturation, which also contribute to the telomerase enzymatic complex [29]. Despite extensive research on these proteins, the three-dimensional structure of human telomerase is yet to be fully understood [30]. Importantly, only h*TERC* and h*TERT* are necessary for the reestablishment of telomerase activity [31–33].

h*TERT* mRNA expression is strictly controlled and closely associated with telomerase activity, which suggests that h*TERT* is the primary determinant for the enzyme activity. Current knowledge proposes that the limiting factor for telomerase activity is h*TERT* expression which is tightly regulated at transcriptional level [34–37]. Experimental evidence suggests that telomerase activity shows strong association with h*TERT* expression [30]. h*TERC* acts as a template for the synthesis of telomeric DNA, and unlike h*TERT*, is ubiquitously expressed in all tissues. Therefore, it has been considered by some authors as a non-limiting factor of telomerase activity [38, 39]. However, another study performed in fibrosarcoma-derived HT1080 cells [40] revealed that h*TERC* is more abundant in tumors than in normal cells with its locus amplified, and is essential for telomerase activity and can be a limiting factor [40].

h*TERT* regulation is a multifarious process yet to be fully understood where both transcriptional and posttranscriptional mechanisms are involved [38]. These include pre-mRNA alternative splicing of the h*TERT* gene which was found to be involved in the regulation of telomerase activity [41–43] and has been associated with diagnosis, prognosis and clinical cancer parameters [43].

### h*TERT* regulation in normal cells

Telomerase is constitutively activated in germline, hematopoietic, stem and also rapidly renewing cells [44, 45]. On the other hand, telomerase activity is very low or absent in somatic cells mainly due to tight h*TERT* regulation [46]. However, telomerase activity was found in normal human blood cells and other normal human cell types that are mitotically active, such as proliferative basal skin layer, endometrial tissue (during menstrual cycle), proliferative zone of intestinal crypts, and hair follicles [44, 45, 47–52].

Telomere length and telomerase activity diverge between normal and embryonic stem cells. While embryonic stem cells fully maintain their telomeres and exhibit telomerase activity, normal stem cells have progressive telomere shortening and minimal telomerase activity (Fig. 1). Since h*TERT* is not expressed in most normal human cells, it can be used as a potential cancer biomarker. In fact, there are studies suggesting that telomerase activity might be a useful marker for diagnosis (detecting cancer disease) and prognosis (associated with stage and disease outcome) in different cancers (e.g., prostate, bladder, thyroid, breast, colon, gastric and lung) [53–65].

### h*TERT* regulation in Cancer

Cancer arises when normal cells accumulate genomic instability and acquires limitless proliferative capacity [66]. Cancer cells have acquired the ability to overcome their fate of senescence via telomere length maintenance mechanisms, mainly by telomerase activation or alternative mechanisms (alternative lengthening of telomeres – ALT) [3, 67–69]. In 1994, it was shown that telomerase is upregulated in up to 90% of malignancies, and is crucial for oncogenesis and disease progression [68, 70–74].

h*TERT* regulation mechanisms have been studied for the last 20 years, and recent advances mainly related to the discovery of h*TERT* promoter mutations have given new impetus to better understand the mechanisms involved in h*TERT* regulation [75].

However, other alterations were recently reported, and h*TERT* expression is also up-regulated in tumors via multiple genetic and epigenetic mechanisms including h*TERT* amplifications (3%), h*TERT* structural variants (3%), h*TERT* promoter mutations (31%) and epigenetic modifications through h*TERT* promoter methylation (53%) [72, 76].

### h*TERT* regulation in cancer: genetic mechanisms

#### h*TERT amplifications*

Gain or loss of genetic material occurs frequently in cancer where gene amplification is an important mechanism for the oncogenic process. Gene amplification results from a copy number increase associated with overexpression of the amplified gene. Different models have been proposed for the initiation of amplification including DNA replication errors, telomere dysfunction and the existence of chromosomal fragile sites [77]. Specifically, h*TERT* gene amplification can result from telomere dysfunction in addition to breakage at fragile sites and formation of chromosomal fusions [78]. In a large cohort made of 31 different types of cancer, it was demonstrated that 3% out of 95% of h*TERT* expressing tumours presented h*TERT* amplifications [76]. Therefore, h*TERT* might be a target for amplification during tumorigenesis, which contributes to the dysregulation of telomerase activity that usually occurs in human tumors [79].

### h*TERT amplifications: clinical relevance*

Increased h*TERT* gene copy number is associated with up-regulation of h*TERT* expression, related to acquired drug resistance, and correlated with worse clinical outcomes in breast, skin and thyroid cancer [79–82]. However, in bladder cancer, no correlation was observed between increased h*TERT* gene copy number and h*TERT* mRNA, telomerase activity, or telomere length, suggesting that h*TERT* gene amplification may require another companion alteration for telomerase reactivation [83, 84].

#### *TERT* genomic rearrangements

Another potential mechanism of h*TERT* upregulation in tumors are the genomic rearrangements affecting the h*TERT* gene locus (5p15.33). Functionally, these rearrangements bring active enhancers in proximity to the h*TERT* gene, and the interaction between the promoter and these newly introduced enhancers drives h*TERT* expression [85, 86]. h*TERT* rearrangements were associated with increased h*TERT* expression, with poorer patient outcome, and found along with other telomere maintenance mechanisms including ALT and MYCN amplifications in neuroblastoma [87].

Further studies are essential to understand whether or not h*TERT* rearrangements are used by different types of cancers, and as well their clinical impact.

#### TERT promoter mutations

In 2013, two pivotal studies described two recurrent non-coding mutations within the h*TERT* promoter region in both familial and sporadic melanomas [88, 89]. These two mutations were located at -124 and -146 bp upstream from ATG (chr5:1,295,228 G>A and 1,295,250 G>A, C>T on opposite strand). After the initial discovery, h*TERT* promoter mutations (*TERT*p^Mut^) have been identified in multiple and distinct tumor types, such as glioblastoma, bladder and thyroid cancer, with different prevalence according to cancer type and histology [90].

*TERT*p^Mut^ represent a frequent but unique genetic alteration that drives h*TERT* expression and telomerase activation. h*TERT* core promoter consists of 260 base pairs with multiple transcription-factors binding motifs that regulate gene transcription and telomerase activation [91]. The location of these mutations within the promoter creates additional binding sites for the E-twenty-six (ETS) transcription factor family, thus constituting a novel mechanism of genetic activation in cancer and a possible driver genomic alteration [92, 93].

**Fig. 2:**
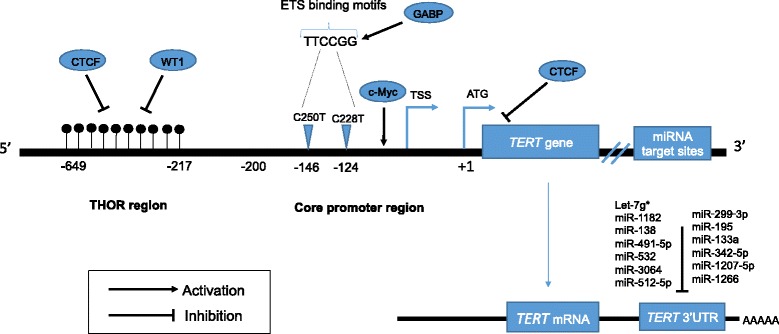
Mechanisms of h*TERT* regulation in cancer. Transcription factors and their binding sites, as well the positions of both h*TERT* promoter mutations, C228T and C250T, the hypermethylated region upstream to TSS (THOR) and TERT-miRNAs are shown. The cancer-specific mutations within the core promoter, at -124 and -146bp positions generate ETS binding motifs, leading to GABP transcription factor recruitment and consequently h*TERT* transcription. Binding of transcriptional activators (c-Myc) and repressors (WT1 and CTCF) to the hTERT promoter may be controlled by DNA methylation, in which methylated CpGs prevent their binding to the target sites, leading to hTERT activation (THOR region). MiRNAs targeting the 3’UTR promotes translation repression of h*TERT*. Black dots represent methylated CpG sites. ETS: E-twenty-six; TSS: transcription start site; ATG: start codon

The transcriptional controlling of h*TERT* gene is complex and includes regulation at multiple levels by various positive and negative factors or pathways. Recent knowledge has come from the cloning of h*TERT* promoter and identification of various transcription factor-binding motifs, involved in h*TERT* expression and telomerase regulation by *TERT*p^Mut^ [22, 30, 39, 94–96]. *TERT*p^Mut^ modulate transcriptional regulation without altering an encoding protein. Functionally, h*TERT* promoter mutations are associated with the formation of consensus binding sequence (CCGGAA) at the E-twenty-six/ternary complex (ETS/TCF) transcription factors (Fig. 2), providing a possible mechanism for cancer-specific upregulation of telomerase [88, 89]. Mechanistically, ETS transcription factor binding to the motifs (created by the mutations) causes a recruitment of a multimeric ETS family member, the GA-binding protein alpha subunit (GABPA) that activates h*TERT* transcription [88, 97, 98]. These findings were further explored through luciferase reporter assays showing increased telomerase activity in cells transfected with mutant constructs. [88, 89, 99] Moreover, there is an evidence of promoter mutations creating *de novo* transcription factor binding sites, as cells co-transfected with mutant promoter constructs and plasmids containing ETS1 cDNA display increased activity [100]. In cancer cells harboring *TERT*p^*Mut*^, the mutant promoter recruits GABPA and exhibits H3K4m2/3, an active chromatin mark. On the other hand, wild type cell lines exhibit the H3K27me3, a mark of epigenetic silencing, suggesting that only the mutant promoters are transcriptionally active [98]. Despite both mutations are functionally active the *TERT*p^Mut^, C228T is significantly more frequent than the C250T [101].

The wide distribution across different tumors (urothelial cancer – bladder and upper urinary tract, melanoma, glioblastoma, thyroid cancer, hepatocellular carcinoma) and high frequency in some of them has created an important hub around these genetic alterations [90, 99, 102, 103]. Bladder, thyroid, cutaneous melanoma, basal cell and squamous carcinoma and oligodendrogliomas are examples of cancers where *TERT*p^Mut^ are widespread through different stages and grades of the disease, suggesting their role as an early tumorigenic event [102, 103]. Additionally, not all *TERTp*^Mut^ tumors display telomerase activation and some premalignant lesions also displayed these genetic alterations at the h*TERT* promoter region [104]. Together, these results support the fact that *TERT*p^Mut^ may act as early events in the oncogenic process [90, 105–107].

Important information came recently from a new study demonstrating that *TERT*p^Mut^ are necessary but not sufficient to maintain telomere length nor telomerase upregulation [108]. In fact these authors demonstrated that *TERT*p^Mut^ acquired at the transition from benign nevus to malignant melanoma do not support telomere maintenance suggesting that *TERT*p^Mut^ contribute to tumorigenesis in two distinct ways. Initially, *TERT*p^Mut^ do not prevent telomere shortening but act “healing” the shortest telomeres and later telomeres are critically short leading to genomic instability and telomerase reactivation [108].

These results might support the hypotheses that *TERT*p^Mut^ are not the unique event responsible to initiate an oncogenic process explaining their presence in premalignant lesions and non-h*TERT* expressing tumors. *TERT*p^Mut^ usually occur in cancers with low rate of self-renewal, such as brain tumors, liver, melanocytes and even low-grade bladder cancers suggesting a role in triggering telomerase activation [109, 110]. In adult gliomas, *TERT*p^Mut^ were found in 70% to 80% of glioblastomas, followed by oligodendrogliomas (60%-70%) and oligoastrocytomas (35%-55%). However, *TERT*p^Mut^ are rare events in ependymoma lesions [110, 111]. Urological malignancies have a different prevalence of *TERT*p^Mut^ varying from rare or absent in prostate cancer and testicular germ cell tumors to high frequency amongst urothelial cancers. In urothelial bladder cancer, mutations are present in up to 85% of the lesions, which rank these alterations as one of the most frequent genomic events in bladder cancer [112, 113]. However, the prevalence in renal cell carcinoma is low, at approximately 9% [106, 111–113]. Regarding thyroid cancer, the frequency of these genetic alterations varies according to histology. Papillary and follicular type lesions usually harbor 10-20% whereas in poorly undifferentiated and anaplastic lesions *TERT* mutations are found in 30-50% of the patients [107]. There is a stepwise increase in frequency of *TERT*p^Mut^ from well differentiated to poorly differentiated lesions in thyroid cancer being absent in medullary carcinomas [107, 114]. However, there are other cancers that do not harbor *TERT*p^Mut^ (testicular germ cell tumors; breast cancer, colorectal carcinoma, prostate cancer) but have telomerase activation [111]. These observations suggest that in h*TERT*-dependent tumors without *TERT*p^Mut^, other mechanisms responsible for telomerase activation might be at play.

### TERT promoter mutations: clinical relevance

Clinically, tumors carrying *TERT*p^Mut^ frequently express higher levels of h*TERT* mRNA and telomerase activity compared with those having a wild type promoter highlighting the prognostic potential of *TERT*p^Mut^ and their potential use as a clinical biomarker [90]. Several studies have looked at the role of *TERT*p^Mut^ in cancer diagnosis and prognosis. In urothelial bladder cancer patients, *TERT*p^Mut^ were detected in tissue and urine and has been proposed as a non-invasive diagnostic and prognostic marker, associated with decreased disease-free survival [102]. However, other studies did not find a clinical correlation with disease outcomes [84, 112]. Wu et al. [115] reported an important co-occurrence of *TERT*p^Mut^ and TP53/RB1 mutations and suggested that they might cooperatively contribute to the progression of bladder cancer.

In glioma, *TERT*p^Mut^ are distributed according to histology and are related to survival in combination with *IDH1* mutations. Also, *TERT*p^Mut^ are not only prognostic factors for poor clinical outcomes, but also predictors of radiotherapy resistance [116–119]. Furthermore, BRAF/NRAS mutations are associated with decreased disease-free and melanoma-specific survival [120, 121]. In liver disease, *TERT*p^Mut^ are present in pre-malignant nodules and predict high risk for advanced disease and reduced disease-free and overall survival in hepatocellular carcinoma patients [122, 123]. Thyroid cancer patients with *TERT*p^Mut^ are associated with clinically aggressive and recurrent disease, with lower disease-free and overall survival when combined with *BRAF* mutations [124–126]. *TERT*p^Mut^ are a moderately prevalent genetic event in non-small cell lung cancer (NSCLC) associated with patient age, gender and distant metastasis [127]. These studies emphasize the hypothetical existence of a companion mechanism, necessary not only for telomerase activation but also to maintain the self-renewal capacity allowing cancer disease progression in *TERT*p^Mut^ patients [84, 112].

Current studies highlight the prognostic properties of *TERT*p^Mut^ and their potential use as a clinical biomarker. In general, these genetic alterations of the h*TERT* promoter are associated with adverse outcomes in several cancers. Nevertheless, recent studies show the presence of companion genetic alterations in patients with worse outcomes, suggesting the need for concomitant and possibly synergistic events resulting in not only telomerase activation but also disease progression.

Unanswered questions remain to be elucidated related to the diverse frequency of mutations amongst different cancers and histological types. Also, the coexistence of h*TERT* regulation mechanisms in the same tumor and the eventual collaborative effects between *TERTp*^Mut^ and other h*TERT* regulatory mechanisms resulting in differential telomerase activation is object for future studies.

### h*TERT* regulation in Cancer: epigenetic mechanisms

#### h*TERT* promoter methylation

The epigenetic process of DNA methylation is crucial in gene expression regulation. DNA methylation occurs genome-wide at CpG sites usually located in non-coding regions. This process, mediated by DNA methyltransferases, occurs in the context of dinucleotide sequence 5’-CG-3’, often referred to as CpG methylation and consists of the addition of a methyl group (-CH_3_) on the 5-carbon of a cytosine (C) base followed by guanine (G) base. CpG dinucleotide sequences are spread throughout the genome, but there are specific regions known as CpG islands where high frequency of CpG dinucleotides is observed. 80% of CpG sites are methylated in intergenic regions while most sites in the promoter and exon 1 regions are unmethylated [128]. CpG islands are usually clustered near the gene promoters where transcription initiation occurs. About 70% of the human gene promoters contain CpG islands, and therefore DNA methylation has been thought to play an important role in gene expression [128, 129]. Promoter DNA methylation has been recognized as one of the most frequent and stable ways of gene expression controlling mechanisms. Hitherto, promoter DNA methylation is thought to promote gene silencing. In actively transcribed genes, the promoter tends to be unmethylated, since DNA methylation has been associated with gene silencing by hindering transcription factor binding or affecting chromatin architecture [130]. In fact, in most cases, genes with methylated promoters are usually silenced while genes with unmethylated promoters are actively transcribed, the pattern observed in oncogenes and tumor suppressors [131]. During cancer progression, there is a genome-wide hypomethylation of CpG sites along gene body and hypermethylation of CpG islands in gene promoter regions [132]. Thus, abnormal DNA methylation is a hallmark of cancer cells and is crucial in cancer development [133].

Despite the powerful role of recurrent h*TERT* promoter mutations in h*TERT* activation in cancers, there are several tumor types that exhibit low or no frequency of these mutations (e.g. prostate and breast cancer) [134]. Thus, the role of epigenetic mechanisms in cancer-specific h*TERT* regulation has been a topic of study for past decade, and several studies have shown contradicting effects of h*TERT* promoter methylation on h*TERT* expression.

Although some authors have reported hypomethylation in the CpG islands covering h*TERT* promoter, others identified increased DNA methylation in h*TERT* expressing cancer cells [135–138]. In fact, h*TERT* was one of the first genes in which methylation of its promoter sequence was positively correlated with gene expression [135]. This correlation among h*TERT* promoter methylation with h*TERT* mRNA and telomerase activity suggests that methylation of h*TERT* promoter may be implicated in h*TERT* regulation, but in a different manner from other genes regulated by promoter methylation [135].

As mentioned above, promoter methylation is often associated with gene silencing. However, several studies have shown that methylation of specific regions within h*TERT* promoter, particularly, upstream of the h*TERT* core promoter, is associated with gene activation [72].

The precise mechanisms by which the methylation pattern of h*TERT* promoter results in h*TERT* activation is still under investigation (Fig. 2). Recently, the possible role of h*TERT* promoter methylation on activation of h*TERT* expression has been functionally shown [72, 139].

There are several explanations as to how h*TERT* promoter methylation can result in h*TERT* activation: first possibility is based on the prevention of repressive elements binding caused by DNA methylation at the repressive region. If h*TERT* promoter is hypomethylated (unmethylated), the transcriptional repressors would bind to the promoter and block the transcriptional machinery (Fig. 2). However, if methylated, h*TERT* would prevent this binding and therefore would allow the promoter to be activated by appropriate transcriptional factors. An interesting observation from these results is that proximal h*TERT* core promoter – allowing essential drivers of gene expression to access the promoter is almost always hypomethylated, and the region upstream of core promoter is often hypermethylated [140, 141]. Whether coincidental or reasonable, recurrent h*TERT* mutations seem to occur in the unmethylated region, which supports the hypothesis stating ETS family factors binding to these sites activate h*TERT* expression. Evidence has been also given by demethylation of repressor binding sites by 5-aza-2-deoxycytidine, globally reducing DNA methylation, and consequently resulting in reduced levels of h*TERT* transcription [142]. Also, factors such as CTCF**,** which interact with h*TERT* promoter, are known for organizing global chromosomal architecture, and methylation-sensitive binding of CTCF may be changing not only the accessibility but also chromosomal conformation and possible interactions with enhancers or silencers far away in distance. CTCF binds adjacent to transcriptional start site (TSS) and represses h*TERT* transcription, but DNA methylation prevents CTCF binding and consequently allows for the activation of telomerase [143].

Wilms tumor protein (WT1) is another repressor of *TERT* expression [144]. WT1 exhibits methylation-sensitive binding to DNA sequence, with reduced binding when one or more methylated bases are present in the binding sequence. WT1 binding sites exhibit increased CpG methylation in cancer, which results in the blocking of repressive effects and consequently h*TERT* expression [135, 136]. MYC proto-oncogene encodes a ubiquitous transcription factor (c-Myc) involved in the control of cell proliferation and differentiation. c-Myc has a direct role in induction of telomerase activity [145]. As CTCF and WT1, c-Myc binding is also methylation-sensitive and its binding is absent or reduced when binding site is methylated, resulting in reduced h*TERT* expression [146].

Another possible explanation is a more complex mechanism involving DNA methylation and chromosome structural changes [147]. DNA methylation can contribute to changes in chromatin conformation influencing gene expression by affecting DNA exposure to transcription factor binding [148]. DNA methylation is often linked to histone modifications and might control the accessibility of transcription factors to the promoter. Specific conformational changes caused by methylation of h*TERT* promoter may be causing differential recruitment and binding of factors that can drive h*TERT* expression in cancer [94]. There are several histone post-translational modifications, such as histone acetylation and methylation, that can affect the compaction state of chromatin, which influences the folding, position and organization of DNA, thereby affecting gene expression [149]. Generally, high levels of H3K4me3 and H3K27ac marks are associated with active chromatin while the gain of H3K9me and H3K27me3 marks has been linked to transcriptional repression [150].

### h*TERT* promoter methylation: clinical relevance

Several tumor types including malignant tumors of brain, prostate, urothelium, colon, and blood have shown high frequency of hypermethylation signature in a specific region upstream of h*TERT* core promoter. More interestingly, even in melanomas – where h*TERT* promoter mutations were first identified and is known to be a mechanism of h*TERT* activation – h*TERT* promoter methylation was associated with h*TERT* upregulation [151]. Despite high prevalence of this tumor-specific signature across various tumor types, there has been little effort put into translating these findings to apply in clinical settings. Methylation of a specific region in the h*TERT* promoter was identified as potential biomarker of tumor progression and survival in pediatric gliomas [72]. This region termed THOR (*TERT* Hypermethylated Oncological Region) is hypermethylated in malignant tumours and hypomethylated in normal tissues and stem cells [72]. THOR is 100% specific and 96% sensitive for detection of h*TERT* expressing malignant neoplasms. THOR methylation showed prognostic properties as well, and identified which low-grade tumours would progress to high-grade ones and predicted survival in a subset of paediatric cancers [72]. THOR was further explored in prostate cancer and has shown its role as a potential marker with diagnostic and prognostic properties [139]. These findings have been expanded upon by multiple groups implicating h*TERT* promoter methylation in h*TERT* upregulation, and further demonstrating not only its diagnostic but, importantly, its clinical significance in cancer prognostic including thyroid cancer, acute myeloid leukemia/myelodisplastic syndrome, esophageal carcinoma, meningioma, pituitary adenomas, colorectal cancer and hepatocellular carcinoma) [72, 82, 139, 152–157]. In these studies, h*TERT* promoter hypermethylation was positively correlated with high h*TERT* expression, telomerase reactivation and in the vast majority of the cases correlated with worse clinical outcomes.

### MicroRNAs

MicroRNAs (miRNAs) are short (20-23nucleotides) endogenous non-coding RNA molecules that have a crucial role in gene expression regulation [158, 159].

The biological importance of miRNAs has been recognized and associated with the pathogenesis of cancer and mechanisms that govern metastatic spread [160]. miRNAs are implicated in genome instability, acting as tumour suppressors or oncogenic drivers. Specifically, miRNAs have been reported to play critical roles in fundamental pathophysiological processes, such as cell proliferation, apoptosis, differentiation and metabolism and present in several human diseases, including cancer [158, 161–165].

Alterations in miRNA patterns in cancer are often associated with genomic events such as mutations, deletions, amplifications and transcriptional changes or due to defects in enzymes involved in miRNA biogenesis. More recent studies however report that epigenetic alterations are crucial regulators of miRNAs in cancer [166, 167]. Functionally, miRNAs mediate the post-transcriptional gene silencing of their target genes, inducing translation repression or mRNA degradation [166]. Downregulation of miRNAs in tumor tissue suggests a tumor suppressor function (suppressor-miRNAs), since a decrease in their expression levels normally contributes to oncogenesis. On the other hand, overexpression of miRNAs that target tumor suppressor genes have been associated with oncogenic activity (onco-miRNAs) [167, 168]. Therefore, depending on their target genes, miRNAs can act as tumor suppressors or oncogenes.

Different miRNAs have been described as important regulators of h*TERT* in multiple types of cancer. h*TERT-*targeting miRNAs regulate negatively its expression, inhibiting tumorigenesis and are frequently downregulated in cancer [167, 169]. h*TERT*-targeting miRNAs biology have been widely studied and their function elucidated through pre-clinical *in vivo* model-based validation studies [164, 170–172]. MiRNAs can regulate h*TERT* in either direct or indirect manner. MiRNAs may directly bind to h*TERT* 3’ untranslated region (3’UTR), and interfere with h*TERT* protein production in cancer cell lines [169, 170, 172, 173]. For example, downregulation of mir-138 was shown to be associated with h*TERT* overexpression in anaplastic thyroid carcinoma cells, and the enforced overexpression of mir-138 induced a significant reduction in h*TERT* expression through interaction with h*TERT* 3’UTR [173]. Additionally, let-7g*, miR-133a, miR-342-5p and miR-491-5p downregulate telomerase activity and inhibit cell proliferation [169]. These miRNAs co-regulate h*TERT* and *Wnt* pathway-genes and importantly, might regulate other genes involved in oncogenesis, suggesting the presence of an oncogenic miRNA regulatory network involving telomerase activation [169, 174–176]. MiR-1182 is other h*TERT* 3’UTR modulator that is downregulated in bladder cell lines and tumor tissues, and whose overexpression was able to inhibit cell proliferation, colony formation, and invasion [171].

MicroRNAs can also regulate h*TERT* indirectly by targeting transcription factors involved in h*TERT* regulation [94]. For example, mir-494 and mir-1294 were reported to downregulate c-Myc, which is a known transcriptional activator of h*TERT,* in pancreatic cancer and esophageal squamous cell carcinoma [94, 177]. Further, miR-34a, a known tumor suppressor in multiple types of cancer, was reported to induce cellular senescence by targeting c-Myc and FoxM1 in the telomere pathway [176].

### MiRNAs: Clinical relevance

MiRNAs are highly stable in a wide range of tissues, including formalin-fixed paraffin embedded (FFPE) tissues and body fluids. These characteristics highlight their use as potential diagnostic and prognostic biomarkers, as well as therapeutic targets [94, 164, 170–172, 178–180]. h*TERT* miRNAs are aberrantly expressed in cancer, and thus constitute a rich source of biological information with high diagnostic and prognostic value. Specifically, miR-1182, miR-1207-5p, miR-1266, miR-532 and miR-3064, which bind within the h*TERT* 3’UTR, are downregulated and associated with a poor clinical outcome in bladder, gastric and ovarian cancer [169–171]. Furthermore, miR-1182 induced chemosensitivity to cisplatin in bladder cancer, and thus, might eventually contribute for a better patient’s response to cisplatin-based chemotherapy [171].

miRNA targeting of genes involved in telomere pathway, might enable telomerase activity suppression and cellular senescence and eventually allow the modulation of other relevant cancer gene pathways, contributing more effectively to inhibit cancer cells self-renewal [181, 182]. Specifically, ongoing clinical research (Phase I, NCT01829971) are testing miR-34a mimics in multiple solid malignancies [182].

Although there is still much to understand about the complexity of telomerase regulation, the discovery of miRNAs that target h*TERT* appears to be a promising approach to prevent and treat cancers that are telomerase-dependent. However, further research is needed in order to provide a more comprehensive view of miRNA-based therapies in terms of delivery systems and toxicity effects and this way promote their translation into clinical reality.

## Future research

Telomerase activation is crucial for cancer development, and was initially thought to be an attractive target for the development of a novel biomarker and anti-cancer therapeutics target [46]. Nonetheless, attempts to inhibit telomerase was devoted to disappointment from the beginning, with the inability of compounds to effectively repress h*TERT* expression and the risk of long-term toxicity to normal stem cells and their self-renewal capacity. Future approaches might be centred on mechanisms responsible for h*TERT* upregulation, as markers for clinical outcomes in cancer. So far, h*TERT* promoter mutations and h*TERT* promoter methylation are strong regulatory alterations that affect telomerase activation and might become useful as potential biomarkers in a wide range of tumors. Moreover, recent studies on ependymomas revealed that the CpG island methylator phenotype (CIMP) tumors, which are associated with poor prognosis, are responsive to drugs that target either DNA or H3K27 methylation [183].

Overall, further research is needed to confirm the potential of these mechanisms as drug-actionable biomarkers, and establish them as non-invasive tools (circulating tumor DNA or circulating tumor cells) with clinical application.

## Conclusion

Cellular self-renewal is a hallmark of cancer which is regulated by telomerase activation, and current studies have shown different mechanisms involved in telomerase regulation. Until recently, telomerase regulation was thought to be controlled uniquely by transcriptional mechanisms. However, different genetic and epigenetic mechanisms have been showing a strong association with telomerase reactivation in different cancers, and importantly showing interesting properties as biomarkers – with diagnostic and prognostic abilities. Particularly, h*TERT* promoter mutations, h*TERT* promoter methylation and miRNAs targeting h*TERT* have gained special attention as mechanisms associated with h*TERT* reactivation. h*TERT* promoter mutations have been frequently identified as early events in tumors with low self-renewal capacity and related to worse clinical outcome. However, several important questions remain to be clarified regarding their role as a tumor initiating mechanism or a long-standing process crucial for oncogenesis and cancer progression. At an epigenetic level, h*TERT* promoter hypermethylation have been positively correlated with telomerase reactivation acting as a predictive marker for oncological outcomes in different cancers. miRNAs targeting h*TERT* have also been considered potentially useful clinical biomarkers, and as more are identified, further avenues for the development of effective cancer therapies are open.

These recent findings generate a spark of hope in advancing our understanding of telomere biology. However, more studies are needed in order to completely understand the complex telomerase regulatory mechanisms and the possible interplay between these mechanisms. Future research should be centred on the discovery of mechanisms responsible for h*TERT* upregulation specifically in cancers, establishing correlations of these biological findings with clinical outcomes and founding these mechanisms as relevant biomarkers. Moreover, h*TERT* regulation remains a very attractive therapeutic target. Understanding the mechanisms responsible for h*TERT* activation might unveil possible means to prevent the acquisition of aberrant self-renewal capacity in cancer cells.
